# The Value of Recreational Physical Activity in Aotearoa New Zealand: A Scoping Review of Evidence and Implications for Social Value Measurement

**DOI:** 10.3390/ijerph20042906

**Published:** 2023-02-07

**Authors:** Kerry Griffiths, Larissa Davies, Catherine Savage, Madeline Shelling, Paul Dalziel, Elizabeth Christy, Rebecca Thorby

**Affiliations:** 1 Sport Industry Research Centre, Sheffield Hallam University, Sheffield S10 2BP, UK; 2 Ihi Research, 518 Colombo Street, Christchurch 8011, New Zealand; 3 Agribusiness and Economics Research Unit, Lincoln University, P.O. Box 85084, Lincoln 7647, New Zealand; 4 Sport New Zealand Ihi Aotearoa, P.O. Box 2251, Wellington 6140, New Zealand

**Keywords:** literature review, Māori, indigenous populations, sport, physical activity, social outcomes, health, wellbeing, social capital

## Abstract

Internationally, there is rising interest in measuring the value of sport and physical activity to society. A critical step in valuing the sector is first establishing the relationship between engagement in sport and physical activity and the societal outcomes that ensue. This paper summarises the findings of a literature review carried out as part of a larger study on the Social Return on Investment (SROI) of recreational physical activity in Aotearoa New Zealand. The review aimed to synthesise existing evidence on the relationship between recreational physical activity and wellbeing outcomes for all New Zealanders, including tangata whenua (Māori, who are Aotearoa New Zealand’s Indigenous population). The methodology took the format of a scoping review and included a series of searches for academic and grey literature, including literature concerning Māori that might have been overlooked in a traditional academic search. The findings are grouped into five outcome areas: physical health; subjective wellbeing; individual development; personal behaviour; and social and community development. The review found some compelling evidence which shows examples of the links between sport and physical activity and outcomes in each of these areas for specific population sub-groups. In particular, for Māori, the findings demonstrate a strong impact on social and community development through building social capital and enhancing cultural identity. However, in all outcome areas, there is mixed quality evidence, a small amount of evidence on which to base definitive conclusions, and limited evidence relating to the monetary value of outcomes. The review concludes that there is a need for further research to strengthen the evidence base for social impact measurement, particularly around the impact of sport and physical activity for indigenous populations.

## 1. Introduction

Globally, there is growing interest in measuring the wider contribution of sport and physical activity to society across the academic community, government, and industry [1,2]. This is reflected in the growth in evidence on the contribution of sport and physical activity to social outcomes, including improved health, life satisfaction, better social connections, higher levels of social inclusion and trust, and better community engagement [3,4]. Moreover, there has been a rise in studies commissioned by various organisations to value and justify investment in this sector [5,6,7,8].

A critical first step in measuring and valuing sport and physical activity is understanding its relationship to social outcomes. Research on the social impacts of sport and physical activity dates back to the 1960s, including extensive studies of outdoor recreation in the USA and later, publicly funded studies on leisure and quality of life in the UK [4]. However, until more recently, the notion that on balance, sport and physical activity create a wide range of social benefits was contested [9]. There is now international consensus that participation in sport and physical activity helps to treat and prevent noncommunicable diseases such as heart disease, stroke, type 2 diabetes, various cancers, dementia, and depression [10]. Furthermore, an increasing number of higher-quality studies show that sport and physical activity are positively associated with other social outcomes such as improved subjective wellbeing, reduced crime, and better educational attainment [11,12,13,14]. Although the quality of evidence for these outcomes is not as robust as for health, the weight of evidence suggests growing support for these wider impacts.

Within the entire body of literature discussed, little attention has been given to the impact of sport and physical activity on people of different cultures and in particular Indigenous communities. For example, Eigenschenk et al. [2], in a recent review on the benefits of outdoor sports for society, grouped the effects of outdoor sport into the categories of physical health, mental health and wellbeing, education and life-long learning, active citizenship, crime reduction, and anti-social behaviour. They highlighted that outdoor sports could have benefits for different ages, people from different financial backgrounds or with special needs, and those in urban areas as well as those from rural areas. However, differences in the impact of outdoor sport for different population sub-groups were not addressed.

The literature review presented in this paper was conducted as part of a wider study on the Social Return on Investment (SROI) of recreational physical activity in Aotearoa New Zealand. The study was undertaken on behalf of Sport New Zealand Ihi Aotearoa (hereafter referred to as Sport NZ) as part of its programme of work to better understand, demonstrate, and communicate the contribution of recreational physical activity to the wellbeing of people living in Aotearoa New Zealand, including its Indigenous population. The review aimed to answer two questions: firstly, what is the relationship between recreational physical activity (including both participation and volunteering) and wellbeing outcomes; secondly, what is the monetary value of wellbeing outcomes related to recreational physical activity? The primary focus of the review was literature relating to wellbeing outcomes for all New Zealanders, including tangata whenua (the Māori population, who are Aotearoa New Zealand’s Indigenous population).

### 1.1. Valuing Sport and Recreation in Aotearoa New Zealand: Review Context

Research on the value of sport in Aotearoa New Zealand has historically focused on economic value. Estimates of the value of sport and recreation to the New Zealand economy date back to the early 1990s and the work of Jenson et al. [15]. Since then, several studies have been commissioned (e.g., [16,17,18]), the most recent being Dalziel’s study on the economic value of sport and outdoor recreation in 2013/2014 [19]. The primary focus of these studies is the measurement of economic value using traditional ‘market’ indicators such as Gross Domestic Product (GDP), employment, and household spending. Since those early studies, public policy in Aotearoa New Zealand has been reframed using a more explicit focus on wellbeing. This is reflected, for example, in the New Zealand Treasury’s Living Standards Framework, which identifies 12 domains of current wellbeing [20].

The most recent and relevant research on the Value of Sport, published by Sport NZ [21], is comprised of three stages: firstly, a review of international literature concerning the benefits from participation in sport and active recreation; secondly, qualitative research with members of the general public and sport and recreation sector stakeholders to gain perspectives on the value of sport; and finally, quantitative research in the form of a survey involving a sample of the general public, people working in the sport and recreation sector, representatives of organisations operating in the sport and recreation sector, and other organisations. This study is an important piece of work and demonstrates the value of sport for achieving wellbeing outcomes in Aotearoa New Zealand to some extent but does not give a complete picture. The findings are based on a mix of evidence and opinion, the research does not monetise the value of sport, and importantly the study does not consider the value of physical activity for Māori. It is against this backdrop that the review presented in this study and the wider SROI study of recreational physical activity in Aotearoa New Zealand were commissioned.

### 1.2. Defining Recreational Physical Activity

There is no standard definition of ‘recreational physical activity’ used by Sport NZ or other stakeholder groups. For the purposes of this review, the research team agreed on a broad definition of recreational physical activity, as follows:

*Competitive sport, undertaken in an organised structure, for example, in a competition or tournament, or informally outside an organised structure, and non-competitive active recreation for enjoyment and wellbeing that occurs in the built, landscape, and natural environments. This may include activities such as kapa haka* (kapa haka—(noun) concert party, *haka* group, Māori cultural group, Māori performing group. Definition from Te Aka Māori Dictionary https://maoridictionary.co.nz/ accessed on 2 February 2023)*, fitness/exercise, dance, tramping, outdoor recreation, and active play but excludes household activities such as gardening and other domestic activities. Active transport for work commuting was also excluded.*

All activities falling within this definition were included within the scope of the study.

This paper is structured as follows. First, it outlines the review method, and then it presents the thematic findings. The paper then summarises the key themes, discusses some methodological limitations, and concludes by examining the implications for the SROI of recreational physical activity in Aotearoa New Zealand and social impact measurement more generally.

## 2. Review Methodology

The methodology used for this literature review was the ‘quick scoping review’ approach described by Collins et al. [22], in which the aim is to identify the evidence available, summarise the findings, and provide an informed conclusion on the volume and characteristics of the evidence base. This was deemed to be the most appropriate method given the need for some flexibility in order to explore the literature available on Māori, along with the timeframe and budget that were available for the review.

The review team comprised experts on SROI and literature reviewing, including Māori researchers based in Aotearoa New Zealand. The review protocol was developed in conjunction with Sport NZ to ensure that the search would uncover the widest range of literature possible, as well as incorporate Māori papers, including papers written in Te reo Māori (Māori language), as well as papers written by Māori researchers and/or Māori organisations.

The starting point for this work was to review the 2017 Value of Sport research by Sport NZ [21], which had included an international literature review on the outcomes of sport and was deemed to be the most relevant piece of research produced in NZ previously. The review then searched for relevant literature produced since that research (that is, from 2017 onwards), as well as any literature on Māori which might be overlooked in a standard academic and grey literature search.

The search involved a range of online academic databases, including Scopus, Web of Science, PubMed, and Google Scholar, supplemented with targeted searches for unpublished grey literature by scanning websites including governmental sources and a variety of sports organisations in Aotearoa New Zealand, including Māori organisations. The searches looked for the combinations of search terms listed in Table 1 that appeared in the title, abstract, or keywords of papers.

After this initial search, a discussion was held between the review team and Sport NZ around their current understanding of the impact of sport and physical activity, in part based on their Value of Sport research [21], as well as the findings of previous international reviews including Taylor et al. [4] and Sport England’s Sport Outcomes Evidence Review [23]. The outcomes of previous work were mapped against the New Zealand Treasury’s Living Standards Framework, in anticipation that the literature would cover outcomes associated with seven of the twelve domains of wellbeing from this framework:

1. Civic engagement and governance;
2. Cultural identity;
3. Health;
4. Knowledge and skills;
5. Safety;
6. Social connections;
7. Subjective wellbeing.

This led to a further round of searches using terms developed and agreed by the review team, presented in Table 2 below.

All literature found from the searches was initially screened by the reading of abstracts, executive summaries, or introductions. Papers deemed relevant to the review were downloaded and read in full. Relevant characteristics from each paper (for example, population, methodology used, main findings, and outcomes evidenced) and data were extracted from these papers into a specially designed spreadsheet which grouped the papers into themes. There was considerable crossover with the papers uncovered under each of the outcome areas presented in Table 2 above; therefore, the papers were further grouped into five overarching themes: physical health; subjective wellbeing; individual development; personal behaviour; and social and community development.

The data extraction process also included an assessment of the volume of literature available under each of the outcome areas, as well as an assessment of the quality of that literature. Whilst in Collins et al. [22], critical appraisal of the literature is not an essential part of a quick scoping review, the review team did make some assessment of the quality of the literature, using a framework based broadly upon the Critical Appraisal Skills Programme (CASP) [24] criteria, in which the following questions were asked:

1. Is the study methodologically sound? (e.g., use of appropriate methods, sampling, recruitment);
2. Are the results of the study valid? (e.g., use of appropriate and clear methods of analysis);
3. What are the results? (e.g., precision of results, confidence in results, reliability)
4. Will the results help locally? (e.g., applicability to the population.)

A traditional CASP assessment would score each paper based upon these categories. However, the assessment made for this review used these questions to provide broad and consistent judgments on the quality of papers but without providing scores. For the wider SROI study, there was a particular emphasis on literature which provided quantitative evidence and any literature which placed a value on the impact of sport and recreational physical activity. Nevertheless, the review included in the scope all literature around the outcome areas, including studies which used quantitative or qualitative methods or examined ‘subjective’ impacts, to enable a full picture of the existing evidence base to be provided. Indeed, the review uncovered a large number of papers which were based on small-scale qualitative studies with a small sample size which explore subjective impacts. These papers might not provide representative evidence for whole populations, but the volume of studies telling a similar story should not be overlooked, and indeed, as this paper will go on to show, the evidence for Māori was typically based on these types of study. For example, the review includes papers which are based on traditional Māori Kaupapa approaches to research, an approach in which research is culturally aligned (conducted by Māori, with Māori, and for Māori), and based on the building of relationships with participants to gather and analyse evidence.

## 3. Results

The following sections outline the findings from the review. As noted above, the crossover in the papers and across outcome areas meant that these findings were grouped into five key outcome areas: physical health; subjective wellbeing; individual development; personal behaviour; and social and community development. For each outcome area, the following sections summarise what the literature demonstrates about the impact of sport and recreational physical activity, any gaps in existing knowledge, and some assessment of the overall quality of the relevant literature.

### 3.1. Physical Health

The evidence around the impact of sport and recreational physical activity on physical health is generally of a higher quality than that found in the other outcome areas, based on more rigorous and robust methodologies. This is at least partly due to health outcomes being more easily quantified than the other outcome areas. The identified papers include five assessments of the monetary value of the impact of sport and recreational physical activity on physical health outcomes, although these are mixed in scope and only two of these papers are based on the impacts solely for Aotearoa New Zealand. The findings for physical health are summarised in the following three subsections, and details of the papers can be found in Table A1 in Appendix A. The first covers papers on the impacts on chronic diseases in terms of prevention or delay of illness. The second addresses the impact on a range of different physiological health markers for older adults, suggesting some positive impacts on healthy ageing. Finally, the third subsection covers papers which estimate monetary costs on the impact of sport and recreational physical activity on health outcomes. It should be noted that in contrast to international literature, there is a lack of New Zealand evidence relating to mental health outcomes, beyond brief anecdotal evidence around some self-reported impacts on stress and anxiety.

#### 3.1.1. Chronic Disease and Illness

There is some evidence to show physical health benefits in terms of prevention or delay of chronic illness for both adults and children. The Value of Sport review [21] reports on (international) literature that identifies relationships between physical activity and reducing type 2 diabetes, high blood pressure, cardiovascular disease, and obesity-related disorders. The literature highlighted by Sport NZ in the Value of Sport review also shows that regular physical activity results in similar outcomes for children, including improved cardiovascular fitness, decreased risk of type 2 diabetes, improved bone health, and maintaining a healthy weight. In addition, there is at least moderate evidence of physical activity having beneficial impacts on rates of breast cancer, colon cancer, osteoporosis, and stroke.

The review finds two further studies [25,26] demonstrating some positive impacts of sport and recreational physical activity on chronic or long-term disease (including helping obese or overweight people to lose weight and address other related cardio-metabolic risk factors), although these are all based on small sample sizes and demonstrate a need for further research to provide evidence on these impacts. In addition, a systematic review by Sushames et al., exploring the literature on the effects of physical activity interventions for Indigenous people in Australia and New Zealand on activity levels and health outcomes, includes 13 studies [27]. Interventions include individual and group-based exercise programmes and community lifestyle interventions. Six studies assess physical activity via subjective (*n* = 4) or objective (*n* = 2) measures, with significant improvements in physical activity levels shown in one study. Weight and BMI are assessed in all but one study, with significant reductions reported in seven of twelve studies. All five studies that used fitness tests report improvements, as do four out of eight measuring blood pressure and seven out of nine in clinical markers. The review concludes that there is no clear evidence for an effect of physical activity interventions on activity levels, but there is evidence of positive effects on activity-related fitness and health outcomes.

#### 3.1.2. Healthy Ageing

There is a small amount of evidence on healthy ageing with just two studies demonstrating the benefits of sport and physical activity for older adults. These include a control group study by Campbell et al. [28], which shows evidence of positive impacts on balance and strength resulting in reduction of falls. Secondly a smaller scale study by Boyes [29] finds that older adults in their study reported physical health benefits in the form of improved sleep, prevention or delay of illness, improved functional ability, reduced chance of falling, being stronger with better endurance, having better flexibility, and better balance and co-ordination.

#### 3.1.3. Monetary Value of Physical Health Outcomes

The identified papers include four assessments of the monetary value of the impact of sport and recreational physical activity on physical health outcomes, although these are mixed in scope. Firstly, Garrett et al. [30], through a systematic review of international literature, report that most interventions to increase physical activity in their sample were cost-effective, especially where direct supervision or instruction was not required.

The other papers found offer an estimate of the impact in terms of health and economic gains, should physical activity targets be met. The studies differ though, in the target population and measures used to monetise impact. Mizdrak et al. [31] estimate the gains that would accrue over the lifetime of the 2011 New Zealand population, if the WHO Global Action Plan for Physical Activity (GAPPA) targets were met. Rush et al. (2014) [32] estimate the expected increases in Quality Adjusted Life Years (QALY) due to avoidance of obesity-related health conditions, and the reductions in health treatment costs, as a result of primary school pupils participating in the Project Energize Programme. However, this study does not isolate the impact of increased physical activity, as this is combined with healthy eating in the research design.

Finally, a report by Waka Kotahi, New Zealand Transport Agency [33], identifies three types of health-related costs attached to physical inactivity, which would be avoided by meeting physical activity guidelines. First, health life quality and expectancy values are estimated from two different methods: disability life years and morbidity and mortality costs. These values are related to health conditions with clear evidence of benefit from physical activity, including heart disease, stroke, two types of cancer, and depression. Second, the direct health system costs are taken from a study of inpatient data from the Ministry of Health (Market Economics [34]). Third, the same Market Economics study reports on lost output cost arising from physical activity. This Waka Kotahi report therefore offers the most comprehensive evidence of the health-related costs of physical inactivity, i.e., the value of physical activity, using New Zealand data.

### 3.2. Subjective Wellbeing

This section brings together papers based on individual’s self-evaluations (including thoughts and feelings) around their more general wellbeing, including satisfaction with life and various ‘softer’ or more holistic impacts. Relative to the volume of papers demonstrating the positive impact of sport and recreational physical activity on markers of physiological or physical health, there are a reasonably high number of papers describing these types of subjective wellbeing benefits. Subjective wellbeing recognises the diverse context and culturally dependent ways of conceptualising wellbeing and the importance of prioritising what people experience [35]. It should also be noted that the majority of papers found on these types of subjective wellbeing outcomes focus on Māori communities (six out of eleven papers). Two New Zealand papers assign a monetary value to subjective wellbeing [36,37]. Summaries of the papers can be found in Table A2 in Appendix A.

#### 3.2.1. Holistic Wellbeing and Māori Culture

Papers focused on Māori communities typically emphasise it is not simply that participation in sport and recreation helps to develop positive feelings of wellbeing, but the whole experience of sport and physical activity is built around Māori culture, cultural identity, spirituality, and the social connections within the Māori community, all of which foster a more holistic sense of health and wellbeing [38,39,40,41]. Consequently, there are many links between the findings in this section and those in the later section on the outcome area of social and community development.

Akbar et al. [38] report on a systematic review to explore qualitative research around the health and wellness impacts of ‘traditional physical activities’ on Indigenous youth from Canada, USA, New Zealand, and Australia. Traditional physical activities include traditional games such as lacrosse, canoeing, and ceremonial dances but also subsistence activities, such as hunting, fishing, gathering and preparation of foods, and cultural activities. In total, this search identified nine studies. The findings are synthesised using an integrated Indigenous–ecological model, which broadly captures health and wellness impacts under intrapersonal, interpersonal, organisational, community, and policy-level outcomes. Across studies, largely spiritual, emotional, mental, and some physical benefits of traditional physical activity were expressed by youth, which consistently highlighted the importance of participation in these activities for holistic health and wellbeing. Youth described participation in these activities as holistically healthy, a way to experience connection to land, ancestry, and community, and a means to develop healthy habits. The review demonstrates the importance of familial and communal relationships that affect these experiences. The authors report that, in general, there is a lack of research qualitatively examining the perspectives of Indigenous youth experiences in such traditional activities.

Rangi [39] explores the traditional beliefs and values of Māori towards physical activity, with the aim to provide insight into the motivations of Māori communities to be physically active. Data were collected through semi-structured interviews with key informants, and traditional data sources such as pūrākau (myths, ancient legends, stories) and whakatauākī (proverbs, significant sayings) were analysed. Four key themes of traditional Māori physical activity are identified. The theme ‘He Māori te noho’ identifies the characteristics of traditional Māori society as a time when Māori defined beliefs, values, and practices were dominant. ‘He Māori te āhua’ is another theme which identifies that a Māori paradigm and worldview meant that traditional physical activity was a part of a broader holistic system of wellbeing. The theme ‘He māori te taiao’ describes the innate relationship Māori held with nature. Lastly, the theme ‘He Māori i tāmi’ demonstrates the impacts of colonisation on traditional Māori physical activity. In general, traditional Māori physical activity was characterised by Māori having tino rangatiratanga (sovereignty, autonomy) and mana motuhake (authority, mana through self-determination) over their lives, an underpinning of mātauranga (Māori knowledge) and Māori values, and wairuatanga (spirituality) that connects the physical practice to a spiritual experience.

#### 3.2.2. Measures of Wellbeing

The review found some studies which use some more objective measures of experienced wellbeing or more specifically described wellbeing benefits. These include Richards et al. [42] who find that those people who met the global physical activity recommendations had 51% higher odds of having a healthy mental wellbeing and scored better on the WHO ‘at-risk’ threshold that indicates further mental health assessment is required. Further studies have found that older adults self-report improved cognitive performance, better memory, feelings of control, positive effects on negative emotions, enjoying life, delaying ageing, and life satisfaction [29]; and women report increased self-efficacy and positive body image [43].

It is arguably more difficult to assign a monetary value to more subjective wellbeing outcomes, as the scientific data linking the outcome with activity are often less robust. However, two studies by Simetrica Jacobs provide monetary values based in New Zealand [36,37]. These studies utilise an instrumental variable approach to value wellbeing, which identifies the income equivalent of the utility gain (or loss) that sport participation bestows on individuals. The first paper is a methodological note [36] which identifies wellbeing values for different levels of sport and exercise. Second, in what is termed a ‘proof of concept’ in monetising the value of sport to wellbeing, Simetrica Jacobs [37] estimates the effects of sport on several of New Zealand’s Living Standards Framework domains and through these on wellbeing. In addition, the authors estimate the monetary value of the secondary effects of sport on wellbeing for the health domain (i.e., through indirect financial savings), using the New Zealand Treasury’s CBAx model, a form of cost–benefit analysis. However, it must be noted that this proof of concept is not able to use New Zealand data but instead uses UK data. It may therefore not be accurate for Aotearoa New Zealand, and importantly it is not able to differentiate values for different ethnic groups in Aotearoa New Zealand.

### 3.3. Individual Development

In comparison to other outcomes, the volume of evidence found around the impact of sport and recreational physical activity on individual development is small and mixed in quality. Summaries of the papers found can be seen in Table A3 in Appendix A. The papers are grouped into two areas: firstly, papers which highlight impacts upon the development of academic or educational skills or achievement; and secondly, papers referring to the impact upon people’s performance and skills at work or through volunteering.

#### 3.3.1. Educational Outcomes

The review found a low number of papers relating to the impact of sport and recreational physical activity on educational outcomes, and those papers that were found are mixed in quality—often being anecdotal, for example, or based on qualitative research in which people describe that they believe sport and recreational physical activity has an impact, but this is not specifically measured in terms of the measured change in attainment levels, grades, or performance (for example, [44]).

Both the qualitative and quantitative research undertaken as part of Sport NZ’s Value of Sport [21] research find that New Zealanders believe that sport could have educational outcomes through providing a platform for achievement, which helps build confidence, giving young people a sense of worth, pride, and confidence. Whilst this is discussed in relation to achievements in sport, it appears to be assumed that developing these qualities through sport can translate into academic performance. The quantitative survey shows that 88% of respondents believed that sport and other physical activities provide people with opportunities to achieve and help build confidence. In addition, 88% believed that many essential life skills are developed playing sport, including how to interact with others, how to work as a team, how to share, how to compete, how to win, and how to lose. Respondents identifying as Māori were more likely to report a range of personal impacts including life skills. Māori respondents also were more likely than others to report that sport and active recreation had helped their dependents develop important life skills (72%, compared with 59%).

The review found one study by Kulinna et al., [45] which tests the effect of physical activity, in the form of dance, on children’s selective attention in school. It concludes that existing school opportunities focused on cognitively engaging physical activity, such as dance, can improve aspects of students’ selective attention.

#### 3.3.2. Employment and Volunteering Outcomes

The review found one paper relating to the impact of sport and recreational physical activity on work performance. Williden et al. [46] assess individual health behaviours against measures of work performance and find that the impact of psychological distress, physical inactivity, and smoking on productivity suggests that employers might benefit from contributing to health promotion within the workplace.

In terms of volunteering, the review uncovered one paper which provides some insights into the demographics of New Zealand sports volunteers, the capacity in which they volunteer, and their motivations for volunteering [47]. It finds that volunteers aged 24 years or younger are more likely to be motivated to volunteer in order to gain new skills and to improve their employment opportunities. The report does not show, however, what skills are developed or whether or not volunteering does indeed improve employment opportunities as was anticipated.

### 3.4. Personal Behaviour

The papers grouped under the outcome area ‘personal behaviour’ include papers relating to pro-social behaviour, anti-social behaviour, and crime. The review finds that this outcome area is limited in evidence in comparison to the other areas, as well as mixed in the quality of the research found, which reflects the international literature in this area. Outlines of these papers found can be seen in Table A4 in Appendix A.

Three papers [48,49,50] relate to studies based in Aotearoa New Zealand. These show that people believe that sport and recreational physical activity have the outcomes of the development of useful life skills and improving life chances, including through developing good character, team building, goal setting, anger management, and building discipline and self-esteem. The findings show that people believe sport could have a positive outcome for young people in deterring them from anti-social behaviour and ‘keeping them off the streets.’

### 3.5. Social and Community Development

Evidence around the impact of sport and recreational physical activity on social and community development in Aotearoa New Zealand is by far the largest in volume of all the outcome areas studied in this literature review. This differs from previous international reviews in this area such as those by Taylor et al. [4] and Sport England’s Sport Outcomes Evidence Review [23], which have tended to find this to be the outcome area with the least amount of existing evidence in other countries. This finding does not necessarily mean that sport and recreational physical activity have a greater impact on social and community development for people in Aotearoa New Zealand and could simply reflect more academic interest in this area for Aotearoa New Zealand in comparison to other countries. However, it is worth noting that many of the papers in this section relate to Māori communities, which suggests that, for Māori, this is an important outcome of sport and recreational physical activity. There is also considerable crossover with the outcome area of subjective wellbeing, for the evidence suggests that feelings of wellbeing are often developed through the social connections and expression of identity and culture that are built around sport. The papers reported in this section are mostly based on qualitative research demonstrating subjective impacts and self-reporting, which does reflect the finding of previous reviews in this area; for example, the Sport Outcomes Evidence Review in the UK [23] (p. 8) described that social and community development is ‘one of the hardest outcomes to evidence, because the concepts involved-social capital, trust, networks-are notoriously hard to define and measure’. Outlines of the papers found can be seen in Table A5 in Appendix A to this paper.

#### 3.5.1. Bonding Social Capital

Many papers found in this review demonstrate the ways in which involvement with sport and recreational physical activity can develop social connections. This includes through feelings of belonging and inclusion amongst family and friends and enabling people to meet new people and make new friends. In this way, sport can be seen as a form of ‘bonding social capital’, a type of capital that describes connections within a group or community amongst people who have similar characteristics or close relationships. Sport NZ’s Value of Sport report [21], for example, describes how sport and physical activity are perceived as having the potential to strengthen social networks and build a sense of belonging for participants. The cross-sectional survey data found that 52.5% of participants indicated that social reasons were their main reason for taking part. The report also describes that sport brings people within communities together and helps to make friends and develop networks. Seventy-seven per cent of respondents agreed that sport and physical activities help instill a sense of pride in communities. In addition, across all the survey respondents, 73% were of the opinion that sport and physical activities help build vibrant and stimulating communities, but this view was stronger amongst Māori respondents, for whom 80% believed sport and physical activities help build vibrant and stimulating communities.

Papers show positive impacts reported for older adults in the form of social support, making friends, and feeling integrated as a community [29], for young people through developing friendships, a sense of who they are among others (an appreciation of their own strengths and weaknesses), a sense of belonging (when accepted and appreciated by others), and a sense of community (when valued by a team) [26,51]. A further paper by Sengupta et al. [52] demonstrates that sport and recreational physical activity had a significant impact on feelings of community for adults over 18 in its sample.

Three papers explore the importance of sport for family bonding [38,48,53]. These papers show that people believe that sport provides important opportunities for social interaction and the opportunity to meet and socialise with family and community. Portaluri [53] describes how a Māori rugby team behaves as a whānau, a Māori family (widely defined, well beyond a single household), in which ceremonial customs and collective Māori principles are closely observed: reciprocity, support (awhi), hospitality, and unity (kotahitanga) are comprised in rugby camaraderie.

In addition, the papers find that the social connections developed through sport and recreational physical activity in turn lead to feelings of subjective wellbeing. A case study on the growth of the sport of waka ama by Sport NZ [54], for example, describes waka ama (outrigger canoeing) as a major vehicle for Māori cultural identity for both participants and supporters, with culture being deeply embedded in the sport. There has been an increase in waka ama participation in Aotearoa New Zealand in recent years: between 2013 and 2017, it experienced a 34% increase in the number of clubs, a 54% increase in membership overall, and the youth grades saw an increase of 124%. The case study reports that a key benefit of waka ama is health, fitness, and wellbeing (hauora). Feedback from participants shows that waka ama is viewed as a ‘lifestyle’ rather than a sport, and the camaraderie and involvement both on and off the water provides enjoyment and feelings of life satisfaction, with waka ama said to address spiritual, physical, and emotional wellbeing.

#### 3.5.2. Bridging Social Capital

The review identified a small number of papers showing ways in which sport and recreational physical activity bring groups of people or communities together with a common goal or a common action. The literature in this area also demonstrates the potential for sport and recreational physical activity to have an impact on ‘bridging social capital’, which is a type of capital that describes connections that link people across areas that might typically divide society (such as race, class, gender, or religion) or between groups or organisations. Thus, whilst the papers in the section above highlight examples of papers which show sport as having an impact on strengthening social connections (bonding social capital) between groups of family and friends, the papers discussed here show how sport can build relationships between wider groups of people. Indeed, sport is often seen as having the potential to foster feelings of pride, and sports tournaments and events have been acknowledged as contributors to feelings of national identity, a sense of belonging, pride, and feelings of social cohesion. Sport NZ’s Value of Sport report [21] describes some evidence internationally which shows that sport and physical activity can help develop feelings of belonging and inclusion, particularly for migrant populations. In addition, the Value of Sport survey research shows that 83% of participants believed that sport and active recreation at both community and high-performance levels strengthen national pride and feelings of national identity.

The review also finds evidence that sport and recreational physical activity can help foster feelings of community pride, for example, through people taking pride in the successes of their local sports teams, as well as events hosted at community sport facilities and the facilities themselves [49,55]. These papers describe mostly qualitative research to gather perceptions around the impact of sport. Based on research in Australia, KPMG [56,57] calls for further research to quantify the impacts in this area, including measurement of levels of social trust in a community, and this seems to also be a gap in the current evidence for Aotearoa New Zealand.

#### 3.5.3. Cultural Identity

Whilst the papers in the above two sections demonstrate how sport and recreational physical activity facilitate collectivity, bringing people together as a community, both across and within different social groups, the papers outlined in this section demonstrate specifically the ways in which sport and recreational physical activity may foster feelings of cultural identity. In particular, some of the papers explore how the ability to participate in traditional Māori sports, or in sport more generally, ‘as Māori’ can reinforce Māori culture and identity, particularly through the value of whanaungatanga (sense of belonging).

Indeed, a key theme in the literature is the importance of the ability of Māori to participate ‘as Māori’. Participating ‘as Māori’ requires a recognition of Māori understandings of holistic wellbeing (hauora), This is discussed, for example, by Severinsen and Reweti [41] in the context of the sport of waka ama. This paper is based on research with sixteen participants in waka ama, both male and female, who were interviewed through a series of loosely structured conversations, both one-to-one and focus group discussions. Participants were asked about their motivations to join waka ama, what the sport meant to them, and about the social, cultural, and health benefits of being involved in waka ama. The findings from this research show that waka ama provides physical health benefits for participants, but more than this, it provides ‘ora’, meaning ‘to be well’ or ‘total wellbeing’, and the spiritual, social, and environmental benefits are also important outcomes of waka ama. Such benefits are described as just as significant to wellbeing as being physically active.

Further papers are based on case studies of either specific interventions [51,58] or specific sports teams. Work by Hapeta [59,60], for example, shows how incorporating Māori concepts into rugby team strategy and development brings a greater sense of unity and benefits wellbeing (identity and leadership) in the team environment but also in other contexts outside of sport in the wider community.

## 4. Discussion

### 4.1. Summary of Findings

This literature review finds evidence of mixed volume and quality on the extent to which sport and recreational physical activity contribute to wellbeing outcomes in Aotearoa New Zealand. Of the papers found, the largest volume of literature is around social and community development, followed by physical health. For the other identified outcome areas of subjective wellbeing, individual development, and personal development, there are smaller volumes of literature. Despite the varying volumes of existing literature, there is some compelling and useful evidence which does show examples of the links between sport and recreational physical activity and wellbeing outcomes in each of the areas for specific sub-groups, including for Māori communities.

In terms of the quality of evidence available, physical health is the strongest area, and unsurprisingly, the area for which the review finds the most examples of research estimating the monetary value of physical health outcomes. This is at least partly due to health outcomes being more scientifically quantified than the other outcome areas. There is some evidence to show physical health benefits in terms of a reduced risk of chronic disease and illness for both adults and children and a smaller amount of evidence on healthy ageing. Relating to mental health outcomes, there is a lack of New Zealand evidence in the literature beyond brief anecdotal evidence around some self-reported impacts on stress and anxiety. In terms of subjective wellbeing, there is some evidence to show the impact on holistic wellbeing/total wellbeing or ‘hauora’, as well as some evidence around increased life satisfaction and feelings of happiness. The evidence for subjective wellbeing is, of course, based on self-reporting or perception. The papers around individual development are mixed in quality but demonstrate some examples of, in education, positive impacts on self-determination, self-efficacy, cognitive performance, educational attainment, attention, development of life skills, and, in the workplace, increased productivity, although there is a shortage of evidence around employment outcomes. Personal development papers show examples of reduced anti-social behaviour and crime, although these are self-reported, based on anecdotal evidence, and there is a small volume of papers in this area.

The strongest outcome area in terms of volume is around social and community development. A large volume of literature addresses the impacts on developing social capital, including both bonding social capital (through strengthening social connections) and bridging social capital (building connections with diverse groups), and developing cultural identity, in particular for Māori. Several papers show that having an ability to participate in traditional Māori sports, or in sport more generally, ‘as Māori’ helps to reinforce Māori culture and identity. This is an important finding, for previous reviews examining the impact of sport and recreational physical activity have found much fewer papers evidencing the impact on social and community development. This does not necessarily mean that this impact is unique to or more significant for Māori but perhaps more indicates a lack of evidence in this area in other countries or for other populations. It does certainly suggest, however, that this is an important outcome for Māori which should not be overlooked. Indeed, the evidence which suggests that the building of cultural identity and social connections in turn develops feelings of subjective wellbeing through improved life satisfaction or happiness is also an important finding.

It should be noted that, in all of the outcome areas, there is still a fairly small amount of evidence on which to base definitive conclusions, there is a mixed quality of evidence, and there is a lack of New Zealand specific evidence on monetising outcomes. The majority of papers use cross-sectional studies with no investigation of confounding factors. Even in studies covering one outcome area that do use validated and recognised measures, the use of different measures means it can be difficult to make comparisons. There is also a lack of evidence around the impact of recreational physical activity for other minority ethnic populations in Aotearoa New Zealand (for example, Pacific Peoples or Asian populations) across all outcome areas, meaning it is not possible to generalise the results across all members of the population.

### 4.2. Limitations of the Review

A quick scoping review methodology was chosen for this review based on several factors: the aims of the review were to identify and summarise the evidence available; the time and budget available for the review were limited; and the inclusion of Māori evidence meant the review needed some flexibility. The complex nature of the populations being studied meant it would be difficult to use a more precise systematic review method. Nevertheless, multiple searches were undertaken by different members of the review team, and the search was broad with a wide number of search terms used. Whilst this was felt to be the best methodology to meet the aims of the review and to ensure that the review captured relevant Māori literature, it does mean the review is unlikely to be replicable.

In addition, the review included some quality assessment of the literature. Despite this not being an essential part of a quick scoping review methodology, it was deemed to be an important part of the review process in this case, to aid the subsequent SROI study, for there was a need to identify the extent of existing quantitative data and any studies which placed a monetary value on the outcomes of sport and recreational physical activity. The quality assessment, however, was based broadly on CASP principles [24], using the questions posed by CASP but without the scoring of papers. Therefore, the discussions around the quality of the literature are based solely on the (maybe subjective) judgement of the review team.

## 5. Conclusions

This review enhances knowledge and understanding of the relationship between recreational physical activity and social outcomes in Aotearoa New Zealand. It confirms that many of the social outcomes that are associated with engagement in sport and physical activity internationally are also relevant to the general population in Aotearoa New Zealand. In the context of the SROI study and wider social impact measurement, this means that in the absence of New Zealand specific evidence, international evidence may be transferable, particularly in the area of health.

The review also reveals that for the Māori population, there are additional social outcomes that are related to engaging in recreational physical activity that merit attention. In particular, these include the development of social and community connections through enhancing social capital, cultural identity, and providing a holistic sense of wellbeing. Given the lack of robust evidence for these outcomes in Aotearoa New Zealand, there is a clear need for the subsequent Aotearoa New Zealand SROI study to include stakeholder consultations with Māori groups to identify and evidence outcomes. More generally, for social value research which includes Indigenous communities, there is a need to explore more culturally appropriate ways of enabling outcomes to be identified, measured, and valued.

The review presented in this paper serves as a baseline synthesis of evidence on recreational physical activity and social outcomes in Aotearoa New Zealand. It contributes to the literature by being the first such review to incorporate evidence from a Māori perspective but more importantly for highlighting the importance of adopting a bi-cultural approach to research synthesis in the future. The review does point, however, to a need for further evidence to strengthen the conclusions, in particular around the outcomes for Māori and for other population groups in New Zealand. An implication of this review for social impact measurement is the need to engage with Indigenous communities more fully, to ensure their stories of change are reflected in social impact measurement frameworks.

## Figures and Tables

**Table 1:** Initial search terms.

Type of Activity	Type of Engagement	Type of Impact	Geographical Area
Sport Active recreation Recreational physical activity Exercise Physical activity	Participation Volunteering	Social impact Social value Social benefit Social cost	New Zealand (primary focus) International (for comparison)

**Table 2:** Additional search terms to explore outcomes.

High Level Search Terms	Search Terms
Civic engagement and governance	Social capital (bridging)
Cultural identity	Cultural capital Social capital (bonding) Cultural capability Spiritual health
Health	Physical health Mental health
Knowledge and skills	Academic attainment Educational attainment Academic achievement Educational achievement
Safety	Pro-social behaviour Anti-social behaviour Crime
Social connections	Social capital (bonding) Belonging Community Family health Family and friends
Subjective wellbeing	Life satisfaction Happiness Anxiety Worthwhileness

## Data Availability

Not applicable.

## Appendix A. Literature Summary Tables

**Table A1:** Physical health literature results.

Reference	Type of Engagement	Outcome(s)	Demographics	Methods
Sport New Zealand (2017) [21]	Participation in sport and physical activity in general	Range of health outcomes identified in international literature including reductions in risk of type 2 diabetes, high blood pressure, cardiovascular disease, and obesity-related disorders	New Zealand	International literature review Qualitative research with general public (*n* = 42) and other sport and recreation sector stakeholders (*n* = 60) Survey with general public (*n* = 1516), people working in the sport and recreation sector (*n* = 346), representatives of organisations operating in the sport and recreation sector (*n* = 121), and other organisations (*n* = 178)
Maddison et al. (2019) [25]	Participation in a rugby-based healthy lifestyle programme	Improvements in body weight, heart rate, blood pressure, cardiorespiratory fitness, and lifestyle behaviours	Overweight men	Randomised controlled trial; participants were randomised to either the 12-week intervention (*n* = 49) or a control group (*n* = 47)
Shultz et al. (2014) [26]	Participation in a boxing intervention	Moderate change in some cardio-metabolic risk factors but not in body weight or BMI	Obese adolescent males (one Pasifika, two Māori)	Measurement of physiological variables before and after intervention Focus groups with participants and their parents
Sushames et al. (2016) [27]	Individual and group-based exercise programs and community lifestyle interventions of four weeks to two years	No clear evidence for an effect on activity levels, but there is evidence of positive effects on activity-related fitness and health outcomes	Indigenous people in Australia and New Zealand	Systematic literature review including 13 studies
Campbell et al. (1999) [28]	Home-based strength and balance training programme	Improved balance and strength, resulting in reduction of falls	Women aged 80 plus	Randomised controlled trial over a two-year period
Boyes (2013) [29]	Outdoor adventure programme	Improved sleep, prevention or delay of illness, improved functional ability, reduced chance of falling, being stronger with better endurance, having better flexibility, better balance and co-ordination.	Older adults	Semi-structured interviews (*n* = 6) Survey (*n* = 80)
Garrett et al. (2011) [30]	Interventions to increase adult physical activity that were based in primary healthcare or the community	Most interventions to increase physical activity were cost-effective, especially where direct supervision or instruction was not required	Range of adult participants	International systematic review of cost-effectiveness studies based on randomised controlled trials including 13 studies
Mizdrak et al. (2021) [31]	Meeting of physical activity targets	Healthcare cost savings	New Zealand population	Estimation of the gains that would accrue over the lifetime of the 2011 New Zealand population, if the WHO Global Action Plan for Physical Activity (GAPPA) targets were met
Rush et al. (2014) [32]	Participation in the Project Energize Programme	Quality Adjusted Life Years (QALY) and healthcare cost savings	Primary school pupils	Estimation of the expected increases in QALY snf healthcare cost savings due to avoidance of obesity-related health conditions
Waka Kotahi, New Zealand Transport Agency (2020) [33]	Meeting of physical activity targets	Healthcare cost savings	New Zealand population	Health life quality and expectancy values are estimated from two different methods: disability life years and morbidity and mortality costs These values are related to health conditions with clear evidence of benefit from physical activity, including heart disease, stroke, two types of cancer, and depression

**Table A2:** Subjective wellbeing literature results.

Reference	Type of Engagement	Outcome(s)	Demographics	Methods
Simetrica-Jacobs [36,37]	Participation in sport and exercise	Estimation of monetary value on subjective wellbeing	Population wide (but uses UK data)	These studies utilise an instrumental variable approach to value wellbeing, which identifies the income equivalent of the utility gain (or loss) that sport participation bestows on individuals
Akbar et al. (2020) [38]	Participation in ‘traditional physical activities’	Health and wellness impacts including ‘holistic wellbeing’	Indigenous youth from Canada, USA, New Zealand, and Australia	Systematic review to explore qualitative research including nine papers
Rangi (2021) [39]	Exploring beliefs and values towards physical activity	Reflections on motivations to being physically active	Māori	Semi-structured interviews Analysis of traditional data sources such as pūrākau (myths, ancient legends, stories) and whakatauākī (proverbs, significant sayings)
Palmer et al. (2022) [40]	Waka Ama/Outrigger Canoe paddling; codes (Rugby); and events (Olympic and Commonwealth Games)	Māori stewardship, Māori self-determination, Māori equity, and Māori customary practices	Māori	Case studies
Severinsen and Reweti (2021) [41]	Waka ama (outrigger canoeing)	‘ora’—meaning ‘to be well’ or ‘total wellbeing’, spiritual, social, and environmental outcomes	Māori	Interviews and focus groups with sixteen participants in waka ama, both male and female
Richards et al. (2018) [42]	Meeting of physical activity targets	Healthy mental wellbeing; scoring better on the WHO ‘at-risk’ threshold that indicates further mental health assessment is required	New Zealand adults	Survey
Boyes (2013) [29]	Outdoor adventure programme	Cognitive performance, better memory, feelings of control, positive effects on negative emotions, enjoying life, delaying ageing, and life satisfaction	Older adults	Semi-structured interviews (*n* = 6) Survey (*n* = 80)
Walters and Hefferon (2020) [43]	Resistance training	Increased self-efficacy and positive body image	Women from the UK, USA, and New Zealand	Semi-structured interviews with 12 women

**Table A3:** Individual development literature results.

Reference	Type of Engagement	Outcome(s)	Demographics	Methods
Banville et al. (2017) [44]	Health education and physical education at school	Success at school	Fifty Aotearoa/New Zealand nine- and ten-year-old students of various ethnic backgrounds from two elementary schools	Focus groups
Sport New Zealand (2017) [21]	Participation in sport and physical activity in general	Educational outcomes include building confidence, sense of worth, pride. Developing essential life skills, including how to interact with others, how to work as a team, how to share, how to compete, how to win, and how to lose	New Zealand	International literature review Qualitative research with general public (*n* = 42) and other sport and recreation sector stakeholders (*n* = 60) Survey with general public (*n* = 1516), people working in the sport and recreation sector (*n* = 346), representatives of organisations operating in the sport and recreation sector (*n* = 121), and other organisations (*n* = 178)
Kulinna et al. (2018) [45]	Dance	Children’s selective attention in school	Children at one Aotearoa, New Zealand, primary school in Years 5 and 6.	Comparison study with a dance group and comparison group undertaking regular classroom work. Testing of physical activity using accelerometers. Selective attention assessed at pretest and after the comparison/physical education sessions with the d2 Test of Attention
Williden et al. (2012) [46]	Healthy behaviours at work	Work performance and productivity	Adults in New Zealand workforce	Health risk assessments (*n* = 747)
GEMBA (2015) [47]	Volunteering in sport	Motivations to volunteer—to gain new skills and to improve employment opportunities	New Zealand sports volunteers	Online survey

**Table A4:** Personal behaviour literature results.

Reference	Type of Engagement	Outcome(s)	Demographics	Methods
Gordon et al. (2013) [48]	General participation in sport and recreational physical activity	Developing good character, team building, goal setting, anger management, and building discipline and self-esteem	New Zealand Pasifika communities	Eight focus groups Six interviews
Sport New Zealand (2018) [49]	Community development programme—using sport	Increased community engagement, positive community interactions with police, and a decrease in antisocial behaviour.	Glen Innes community with high Māori and Pacifica populations.	Case study
Wheaton et al. (2017) [50]	Surfing—as part of sport for development programmes	Improved life chances, equipping youth with the tools for self-improvement and self-management	Young people in New Zealand	Interviews with programme personnel (*n* = 8)

**Table A5:** Social and community development literature results.

Reference	Type of Engagement	Outcome(s)	Demographics	Methods
Sport New Zealand (2017) [21]	Participation in sport and physical activity in general	Strengthen social networks, build sense of belonging, making friends, develop sense of pride in communities, and national pride and feelings of national identity	New Zealand	International literature review Qualitative research with general public (*n* = 42) and other sport and recreation sector stakeholders (*n* = 60) Survey with general public (n=1516), people working in the sport and recreation sector (*n* = 346), representatives of organisations operating in the sport and recreation sector (*n* = 121), and other organisations (*n* = 178)
Boyes (2013) [29]	Outdoor adventure programme	Social support, making friends, and feeling integrated as a community	Older adults	Semi-structured interviews (*n* = 6) Survey (*n* = 80)
Shultz et al. (2014) [26]	Participation in a boxing intervention	Developing friendships, sense of belonging, sense of community	Obese adolescent males (one Pasifika, two Māori)	Measurement of physiological variables before and after intervention Focus groups with participants and their parents
Smith et al. (2021) [51]	Physical education	Developing friendships, sense of belonging, sense of community	Participants in Movewell—physical education resource developed for Aotearoa New Zealand primary teachers	Not based on research—outline of programme and intended aims
Sengupta et al. (2013) [52]	General sports participation	Social capital through sense of belonging	New Zealand adults	Nationally representative telephone sample of New Zealanders (*n* = 6631)
Gordon et al. (2013) [48]	General participation in sport and recreational physical activity	Developing family and community bonds	New Zealand Pasifika communities	Eight focus groups Six interviews
Akbar et al. (2020) [38]	Participation in ‘traditional physical activities’	Developing family and community bonds	Indigenous youth from Canada, USA, New Zealand, and Australia	Systematic review to explore qualitative research including nine papers
Portaluri (2017) [53]	Māori rugby team	Māori rugby team behaves as a whānau, a Māori family	Participants in the team	Case study/thesis
Sport New Zealand (2019) [54]	Waka ama (outrigger canoeing)	The camaraderie and involvement both on and off the water provide enjoyment and feelings of life satisfaction	Participants in Waka ama	Case study
Dowden and Mitchelmore (2010) [55]	Rugby	Feelings of community pride	Members of a rugby club	Case study of a single rugby club—visits and interviews
Sport New Zealand (2018) [49]	Community development programme—using sport	Developing community pride	Glen Innes community with high Māori and Pacifica populations.	Case study
Severinsen and Reweti (2021) [41]	Waka ama (outrigger canoeing)	Social connections and participating ‘as Māori’—spiritual, social, and environmental outcomes	Māori	Interviews and focus groups with sixteen participants in waka ama, both male and female
Sport New Zealand (2019) [58]	MaraeFit sport and active recreation initiative	Social connections and participating ‘as Māori’	Māori	Case study
Hapeta (2018) [59]; Hapeta et al. (2019) [60]	Rugby	Development of identity as Māori	Māori rugby team	Kaupapa Māori approach
